# A Clinical Lecture

**Published:** 1888-05

**Authors:** Daniel R. Brower

**Affiliations:** Professor of the Diseases of the Nervous System, Didactic and Clinical, in the Woman’s Medical College


### ORIGINAL LECTURES.

### A CLINICAL LECTURE.

BY DANIEL R. BROWER, M. D.,
Professor of the Diseases of the Nervous System, Didactic and Clinical, in the Woman's Medical College.

[Reported by William Whitford.]

POLIOMYELITIS ANTERIOR ACUTA.

This patient is 52 years of age. She says that she had two convulsions in quick succession last June. She tells us she had general malaise and a high fever before the convulsions came on, and after she recovered from the convulsions her left leg was partially paralyzed, and two weeks afterward her other leg became paralyzed. Her health previous to this trouble was good. She is married, but has never borne children. On examination of the limbs we find, with the æsthesiometer, that the tactile sense is normal. We find by the use of these two tubes, one containing hot and the other cold water, that she has normal condition of temperature-sense, and she seems to have also keen perception of pain.

Whatever the trouble is, it does not involve the sensory areas. Examination of the condition of the limbs as to temperature shows that they are colder to the sense of touch than they ought to be in a room of this temperature. Comparison of the temperature of the hands with that of the lower extremities shows that the temperature is considerably below normal.

There is paralysis of certain muscles in this case. She can not make certain movements. Examination of the limbs as to the condition of muscular tissue shows waste; the calf-muscles have almost disappeared. She had large limbs at one time, but now they are small in comparison to what they ought to be in a person of her size.

There is no disturbance of sensation, but impairment of motion (paralysis), depressed temperature, and atrophy of the limbs.

Regarding the history of this patient, I would say that I saw her at the time she entered the woman's hospital. Then there was absolutely no power in the lower extremities. Since that time, by the use of electricity every day, using the galvanic current to a strength sufficient to produce muscular contractions, she has improved. She has also had massage daily, and was given internal specific treatment. There were certain things in connection with this case that pointed to syphilis as the cause of this disturbance in the spinal cord, and under this continued electrical treatment, together with massage and the internal administration of mercury, she has made a great deal of improvement.

The affection is an inflammation of the anterior horns of the cord—poliomyelitis anterior acuta—and the interesting feature about it is this: It has occurred in an adult. It is a disease of the spinal cord exceedingly uncommon in adults. It comes on suddenly, with first a severe chill and fever, followed by convulsions, then paralysis. There is nothing in the character of the chill, the fever, or the convulsions to lead one to suspect that the spinal cord is the seat of the disease. One can only locate it when the resultant phenomena—paralyses—have been found.

This form of inflammation of the spinal cord does not affect the bladder or the rectum. This patient has no discomfort about the chest or head. The disease is limited to the anterior horns of the cord in the lumbar enlargement. The territory involved in this pathological process is small. You see a striking difference in the symptomatology from the cases we showed you a week ago —two cases of locomotor ataxia, paralysis agitans, and one of paraplegia. The striking symptoms of the disease under consideration are paralysis, wasting of the lower extremities, a low temperature of paralyzed limbs, and the absence of sensory disturbance. You see also vaso-motor disturbance manifested in both of these limbs; they are swollen, showing impairment in the circulation of the limbs. That comes from the vaso-motor disturbance that accompanies the disease and which accounts also for the depressed temperature of the limbs.

